# Music Audiences 3.0: Concert-Goers’ Psychological Motivations at the Dawn of Virtual Reality

**DOI:** 10.3389/fpsyg.2017.00800

**Published:** 2017-05-23

**Authors:** Jean-Philippe Charron

**Affiliations:** Departamento de Financiación e Investigación Comercial, Universidad Autónoma de MadridMadrid, Spain

**Keywords:** virtual reality, concert attendance, marketing 3.0, psychological motivations, live music audience, mediated performance

## Abstract

Reviewing consumers’ motivations to attend performances in a continuously evolving social and technological context is essential because live concerts generate an important and growing share of revenues for the music industry. Evolving fans’ preferences and technological innovations constantly alter the way music is distributed and consumed. In a marketing 3.0 era, what consumers do with music is becoming more significant than simply owning or listening to a song. These changes are not only blurring the lines between production and consumption (i.e., co-creation), but also distorting the concept of live attendance altogether. Although mediated performances typically lack presence and authenticity, recent advances in immersive technologies, such as spherical videos and virtual reality goggles, could represent a new form of experiencing live music.

## Introduction

Twenty year ago, Kotler and Scheff (1997) recommended cultural managers to give performing arts a marketing orientation, where target audiences’ needs and preferences are central to all decision making. It appears that putting music fans at the epicenter of marketing strategies has become especially relevant in this digital day and age. In that sense, the evolution of music consumption models paired with recent technological advances now offers audiences new ways to participate in live music never thought of before, including *virtually* attending gigs. Therefore, knowledge about music fans’ reasons and motivations to attend live concerts will extend our understanding of music audiences, a key aspect of arts marketing (Pitts and Spencer, 2008; Colbert and St-James, 2014).

Contextually, understanding concert-goers’ motivations to participate in live performances is increasingly important since attendance represents an essential and growing share of revenues for the music industry and performers alike (Harbi et al., 2014). For example, the Spanish music industry’s incomes plummeted because of digital piracy, the economic crisis, and a cultural sales tax of nearly twice the European average. Still, revenues from live music concerts grew a steady 10% over the last years to reach €194.5M in 2015 (APM and SAGE, 2016).

Today’s ubiquitous access to digital content and social networks has modified consumers’ relation to music in general and live concert participation in particular. This paper intends to review actual and future characteristics of concert attendance under the following triad of factors: concert-going psychological motivations, music-consumption models, and technological innovations.

## Why go to Concerts?

The way cultural goods are produced, distributed, and consumed is constantly evolving based on rapidly spreading technology and shifting consumers’ preferences (Harbi et al., 2014). Accordingly, music consumers are moving away from a music acquisition-based model to an access-based model (Wikström, 2012). Up to recently, the possession of music—even in its digital form stored on electronic devices—was valued by consumers and served as the extension of one’s self (Belk, 2013). However, as Spotify, YouTube, and Deezer are becoming the dominant means of mass music consumption (Marshall, 2015), today’s fans are not so much interested in “owning” a song, neither physically nor digitally^1^. Instead, music consumers now favor access to a large quantity of online content. As Belk (2013) noted, part of the value of digital goods came from the time and efforts required to obtain them. In that sense, accessing music on streaming platforms is easier than ever. Consequently, under the access-based model, a listener’s music playlists may well be more valuable than the actual digital songs (i.e., the dematerialized possession) contained in them, as well as the ability to share such lists (i.e., one’s musical tastes) online. These changing consumer preferences are eased by technological innovations including the digitalization of music, but also the pervasiveness of Internet, the multiplication of content-streaming platforms, and the emergence of social networks.

From a psychological standpoint, the digital revolution changed the importance traditionally given to at least two broadly recognized concert-attending motives (Trocchia et al., 2011; Kulczynski et al., 2016). For one, attending a concert to explore discover new artists has become almost irrelevant. Potential concert-goers are better informed than ever about touring bands, concerts dates, and selected venues. They rely on promotional information as well as expert opinions and word-of-mouth. By facilitating information and content, digital technologies encourage participants to try out new performers and reduces the inherent disappointment or financial risks of attending a concert (Burland and Pitts, 2010; Farrugia and Gobatto, 2010).

Conversely, social motives to attend leisure activities generally stem from two necessities: the desire for interpersonal relations and the need for esteem from others (Beard and Ragheb, 1983). The former mostly refers to the sense of community concert-goers experience when attending a live performance. But, as Bennett (2015) suggests, live concerts also complement participation in online communities such as music blogs and social networks. Regarding the later, achieving status and obtaining the respect and admiration of others drive individuals to attend live music performances (Kulczynski et al., 2016), what Holt (2010) refers to as “self-realization through cultural consumption.” That is, concert-goers can earn bragging rights by showing off their participation in live concerts. Although “I was at Woodstock” has long been a status-enhancement statement, the phenomenon grew in magnitude alongside the rapid development of virtual social networks (Lingel and Naaman, 2012). Social media now allows concert-goers to share photos, videos, and comments with offsite but online friends, who in turn may reward concert-goers with instant feedback such as “likes” (Scott and Harmon, 2016). Accordingly, sharing attendance to live performances via social networks can help define one’s identity by indicating a higher level of authenticity, where concert-going is generally perceived as the “real deal,” a more authentic experience than streaming pre-recorded songs, for example (Cresswell-Jones and Bennett, 2015; Shuker, 2016). However, as Lingel and Naaman (2012) suggested, producing online content during a live performance means trading off present (personal) enjoyment for future (social) gains.

## Music Audiences 3.0

Holt (2010) argues that the value of music relates to factors beyond the music itself, and that “musical practices must be analyzed in the perspective of broader social and technological changes.” In that context, the music industry will keep moving away from ownership and access-based models toward a context-based model (Wikström, 2012). Because of recent advances in digital technologies, context-based models offer consumers the necessary tools to experience music rather than just listening to it. These innovations not only blur the boundary between production and consumption of music (i.e., co-creation), but also between live and mediated performances (i.e., liveness).

Under the service-dominant logic of marketing (Vargo and Lusch, 2004, 2008), co-creation implies that engaged consumers participate in creating and giving meaning to products, services, and experiences. In that sense, co-creation is an integral part of the artistic experience, where audiences engage in cognitive, emotional, and imaginal practices to make sense of the performance (Ramsey White et al., 2009; Colbert and St-James, 2014). Live concerts also symbolize a co-creation resulting from the interaction between performers and attendance, and where the end product is the concert experience (Minor et al., 2004; Holt, 2010). Other forms of co-creation also stem from live performances, including bootleg recordings of live concerts later distributed online via social networks (Farrugia and Gobatto, 2010; Lingel and Naaman, 2012).

Technological innovations also distort the frontier between live and mediated performances. Video-streaming platforms and other web-based applications offer users the possibility to attend “live” concerts online. The question of whether mediated artistic performances can procure a similar experience is causing much debate. On the one hand, some argue that digitally mediated concerts impede “the possibilities for the unexpected, iterative, and expansive experience” (Harper, 2015). In other words, even if digital mediation maintains the time dimension (*now*) of live performances, it ultimately loses its space dimension (*here*). In that sense, regardless of technological developments, live performances retain some elements of uniqueness that cannot be reproduced, such as *being there* (Holt, 2010; Harper, 2015).

On the other hand, fueled by the development of immersive technologies such as spherical videos and VR goggles, virtual concerts are rapidly growing in popularity online. Interestingly, the concept of presence is frequently associated with immersive technologies, where greater levels of immersive quality elicit higher levels of presence, in turn enhancing the effectiveness of a mediated experience (Jung et al., 2015; Cummings and Bailenson, 2016).

Whether live or mediated, the concepts of presence, of being part of something unique and special with likeminded, is manifestly a key component of the experiential nature of concert-going (Brown and Knox, 2016). While greater engagement, participation, and involvement tends to improve customer experience (Ramsey White et al., 2009; Dobson, 2010; Chen et al., 2011; Kemp and White, 2013), such relationships in the *virtual* world remain largely unknown.

## Conclusion

Music consumption in the digital era is currently receiving a lot of attention. This paper contributes to the review of selected psychological motivations to attend live concerts in continuously evolving social and technological environments. Conceptually, the paper has addressed fans motives to participate in live music within two different Internet eras, often referred to as Marketing 2.0 and 3.0. The first section consisted of a reflection on the shifting importance of intellectual and social motivations to attend concerts. The second section entailed the growing relation between music context and consumer experience. A brief reflection was also initiated on the emergence of virtual reality as novel form of mediated performances, and where immersive technologies can improve virtual participants’ perception of *being there*, an important factor for concert-goers.

As recent technological advances now offer audiences new ways to participate in live music, including virtually attending gigs, knowledge about music fans’ reasons and motivations to attend both live and mediated virtual concerts will extend our understanding of music audiences in general. A core question raised in this paper, and one that demands further research, is the extent to which technology can increase participants’ engagement and improve the virtual concert experience in the physical absence of others, knowing that social and musical enjoyments often go together (Brown and Knox, 2016). A second question requiring more research is how future developments in immersive technology, although difficult to imagine for the moment, will affect the virtual concert experience and the demand for live performances. Potential changes in consumer preferences are important for the music industry because the economic potential of virtual concerts is almost infinite.

## Author Contributions

The author confirms being the sole contributor of this work and approved it for publication.

## Conflict of Interest Statement

The author declares that the research was conducted in the absence of any commercial or financial relationships that could be construed as a potential conflict of interest. The reviewer SS and handling Editor declared their shared affiliation, and the handling Editor states that the process nevertheless met the standards of a fair and objective review.

## Footnotes

1 The music industry also supported the transition from an acquisition-based to an access-based model as a mean to fight back digital piracy and its negative effect on pre-recorded music sales. Paradoxically, illegal file sharing generated positive externalities for the music industry, such as growing audiences (Curien and Moreau, 2009; Mortimer et al., 2012) and surging ticket prices (Krueger, 2005).
